# *Obox1* deficiency impairs fertility in female mice

**DOI:** 10.1016/j.fmre.2025.04.008

**Published:** 2025-04-19

**Authors:** Li Wu, Jiacheng Shen, Zhenzhen Hou, Yinli Zhang, Yan Bi, Ran Zhang, Heping Bai, Wen Ye, Kang Chen, Jiang Zhu, Chenxiang Xi, Yiliang Xu, Xiaochen Kou, Yanhong Zhao, Chong Li, Hengyu Fan, Rongrong Le, Yixuan Wang, Xiaocui Xu, Shaohua Xu, Hong Wang, Shaorong Gao, Lan Kang

**Affiliations:** a Shanghai Key Laboratory of Maternal Fetal Medicine, Shanghai Institute of Maternal-Fetal Medicine and Gynecologic Oncology, Clinical and Translational Research Center, Shanghai First Maternity and Infant Hospital, School of Life Sciences and Technology, Tongji University, Shanghai 201204, China; b Institute for Regenerative Medicine, State Key Laboratory of Cardiovascular Diseases, Shanghai East Hospital, School of Life Sciences and Technology, Tongji University, Shanghai 200123, China; c Frontier Science Center for Stem Cell Research, School of Life Sciences and Technology, Tongji University, Shanghai 200092, China; d Life Sciences Institute, Zhejiang University, Hangzhou 310058, China

**Keywords:** Subfertility, Hormone deficiency, Abnormal luteinization, Aberrant steroidogenesis, Oocyte-granulosa cell interactions

## Abstract

OBOX1 is a maternal factor involved in oogenesis and follicle development, yet its specific role remains unclear. Here, we demonstrated that *Obox1* knockout female mice exhibit subfertility, characterized by reduced litter size and impaired ovulation. These oocytes show minimal disruption in early embryonic development post-fertilization. However, *Obox1* deficiency leads to decreased levels of gonadotropins and female sex hormones, especially the luteinizing hormone (LH). Exogenous human chorionic gonadotropin (hCG) administration during superovulation failed to rescue the ovulation defect. Post-ovulation, the ovulation-related genes and serum progesterone levels were significantly reduced in *Obox1*-deficienct ovaries, accompanied by dysregulated steroidogenesis-related gene expression. Transcriptomic profiling of *Obox1* deficient metaphase II (MII) oocytes revealed downregulation of genes involved in mitochondrial energy metabolism and biosynthesis, and upregulation of genes associated with cell transport, transcription, RNA processing, translation. Further investigation revealed that follistatin gene expression was upregulated in both MII oocytes and ovaries of *Obox1* deficient mice, along with increased expression of *Gdf9, Bmp15, Foxl2*, and NOTCH signaling components. These findings suggest that *Obox1* is essential for maintaining hormonal balance and ovulatory function through regulating oocyte-granulosa cell interactions and steroid hormone synthesis.

## Highlights

• *Obox1* deficiency result in reduced litter size and impaired oocyte ovulation.
• Female mice lacking *Obox1* exhibit decreased levels of gonadotropins and sex hormones.
• Superovulated *Obox1* knockout mice display an increased incidence of unruptured follicles.
• *Obox1* may regulate ovarian somatic function through paracrine mechanism.

## Graphical abstract

**Figure:**
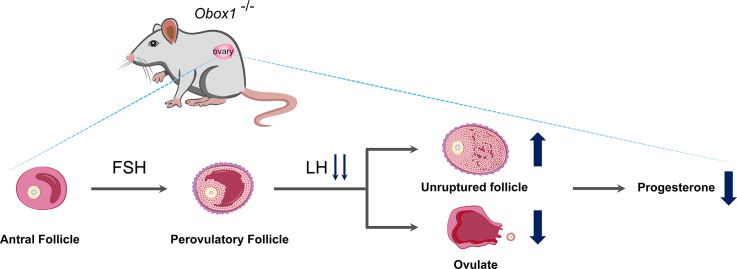

## 1 Introduction

Ovarian follicle, the functional unit of ovary, consists of a central oocyte surrounded by somatic cells, including inner-layer granulosa cells and outer-layer theca cells [1]. Follicular development is orchestrated by the hypothalamic-pituitary-ovarian (HPO) axis, a highly coordinated endocrine system critical for female reproductive function [2]. Gonadotropin-releasing hormone (GnRH), secreted by the hypothalamus, stimulates the anterior pituitary to release two key gonadotropins: follicle-stimulating hormone (FSH) and luteinizing hormone (LH), which act on the ovary to coordinate folliculogenesis and steroidogenesis [3]. FSH promotes oocyte growth, granulosa cell proliferation and estrogen (E2) production [4]. During the follicular phase, rising E2 levels exert negative feedback on the hypothalamus and pituitary, suppressing GnRH and FSH secretion to regulate follicular selection. However, sustained high levels of E2 at mid-cycle induce a surge of LH, which in turn induces ovulation [5,6]. Following ovulation, the ruptured follicle reorganizes into corpus luteum, which produces progesterone (P4) and a smaller amount of E2 to support luteal function and provide negative feedback on GnRH and gonadotropin secretion, maintaining the cyclic rhythm [7,8].

Beyond endocrine regulation, bidirectional communication between the oocyte and granulosa cells is essential for coordinated follicle development [9,10]. Granulosa cells provide metabolic support and signaling molecules to the oocyte through junctional complexes and transzonal projections (TZPs), while the oocyte secretes paracrine factors, such as growth differentiation factor 9 (GDF9) and bone morphogenetic protein 15 (BMP15), which regulate granulosa cell proliferation and differentiation [[11], [12], [13]]. Besides, transcription factors are also essential regulators in oogenesis and folliculogenesis [14,15].

The oocyte-specific homeobox (*Obox*) family genes encode transcription factors predominantly expressed in germ cells, particularly in the ovary [16]. Among them, *Obox1, Obox2, Obox5*, and *Obox7* are highly expressed in oocytes of primary follicles and persist throughout oogenesis, with expression dramatically decreasing at the 2-cell stage [16,17]. *Obox1* and *Obox2* are translated at higher levels than *Obox5* and *Obox7* during oocyte maturation [18,19]. Despite high sequence homology, OBOX2 harbors a frameshift mutation leading to a truncated homeobox domain, potentially compromising its DNA-binding activity [20]. Recent studies have highlighted critical roles of the *Obox* family in mouse zygotic genome activation (ZGA) and early embryonic development [20,21]. Knockout (KO) of multiple *Obox* genes (*Obox1/2/3/4/5/7*) results in developmental arrest at the 2- to 4-cell stage, with *Obox3* shown to regulate ZGA and repeat elements activation in zygotes [20,21].

Our previous work demonstrated that OBOX1 enhances somatic cell reprogramming [17], promoting us to investigate its function in oocyte and early embryonic development. Although OBOX1 is known as an oocyte-specific transcription factor [22], its precise role in oogenesis and folliculogenesis has not been fully elucidated. In this study, we generated *Obox1* KO mice and found that the females exhibited subfertility. Mechanistically, this phenotype may result from increased follistatin expression in oocytes, altered oocyte-granulosa cell communication, disrupted steroid hormone synthesis, and impaired gonadotropin feedback, collectively leading to ovulatory dysfunction.

## 2 Materials and methods

### 2.1 Generation of Obox1 KO mice

All animal experiments were conducted in accordance with the ethical guidelines for laboratory animal care and use at Tongji University (No.TJAB03220105). Mice were housed under standard conditions with a 12-hour light/dark cycle and free access to food and water.

To generate *Obox1* KO mice, a single-guide RNA (sgRNA) targeting exon 2 of *Obox1* was designed using the CRISPR Design Tool. Complementary oligonucleotides were annealed and ligated into the BbsI-digested pX330 plasmid (Addgene 42,230). The sequences of oligonucleotides and primers are provided in Table S1.

The *Obox1* sgRNA and Cas9 coding sequences were amplified by PCR using T7 promoter-linked primers. PCR products were purified by ethanol precipitation and used as templates for *in vitro* transcription with the mMESSAGE mMACHINE T7 Ultra Transcription Kit (Thermo Scientific AM13455). The resulting mRNA was purified again by ethanol precipitation and resuspended in nuclease-free water (Invitrogen 10,977–015).

Purified Cas9 mRNA and *Obox1* sgRNA were mixed, diluted and microinjected into the cytoplasm of zygotes. After 24 h of *in vitro* culture, two-cell-stage embryos were transferred into the oviducts of pseudopregnant ICR females.

Genomic DNA was extracted from tail biopsies of newborn pups using proteinase K (Tiangen RT403) digestion followed by phenol extraction. Gene editing was confirmed by PCR and electrophoresis on polyacrylamide or agarose gels. PCR products were cloned into the pClone007 Blunt Simple Vector (Tsingke TSV-007BS), and individual clones were sequenced to validate the insertion-deletion mutations (indels) in the *Obox1* locus.

### 2.2 Superovulation

To analyze ovulation efficiency, 6- to 8-week-old and 3-week-old female mice, including wild type (WT), *Obox1* heterozygous (Het) and *Obox1* KO genotypes, were subjected to hormone-induced superovulation. Mice were intraperitoneally injected with 5 IU pregnant mare serum gonadotropin (PMSG), followed 46–48 h later by 5 IU human chorionic gonadotropin (hCG). Mice were euthanized at designated time points after hCG injection by cervical dislocation, and MII oocytes were collected from the oviductal ampullae for further analysis.

### 2.3 Oocyte developmental competence assessment

MII oocytes were collected from adult WT or *Obox1* KO female mice (6–8 weeks old) following hormone-induced superovulation. Cumulus-oocyte complexes were mechanically retrieved from the ampulla of the oviducts, and cumulus cells were removed by treatment with hyaluronidase. Mature MII oocytes were then either injected with a single sperm using a microinjector (intracytoplasmic sperm injection, ICSI) or subjected to conventional *in vitro* fertilized (IVF). Sperm used for fertilization were obtained from adult *Obox1* KO male mice.

Following fertilization, zygotes were immediately transferred to embryo culture medium and incubated at 37 °C in a humidified incubator with 5% CO_2_. Embryonic development was monitored at defined time points.

For embryonic stem cells (ESCs) derivation, blastocysts were cultured on mitomycin (MMC)-inactivated mouse embryonic fibroblasts (MEFs). Once outgrowths were observed, the cells were enzymatically dissociated and passaged. The proliferation rate of ESCs was evaluated by manual cell counting.

### 2.4 Histological analyses

Ovaries were fixed in 4% paraformaldehyde (PFA) in phosphate-buffered saline (PBS, pH 7.4) for 12–16 h, then washed with 70% ethanol and stored until further processing. Fixed ovaries were embedded in paraffin, sectioned at 6-μm using a microtome, mounted on glass slides, and stained with hematoxylin and eosin (H&E) for histological evaluation.

### 2.5 Enzyme-linked immunosorbent assay (ELISA) analysis for hormone measurement

To assess hormone levels during diestrus, the estrus cycle stages were determined by vaginal cytology. Vaginal smears were prepared by collecting secretions and examining cellular morphology under a light microscope. Diestrus identified by the predominance of small neutrophils in the vaginal smear. Serum and ovaries were collected at this stage for subsequent hormone analysis.

To evaluate P4 levels during the luteal phase, serum was collected from adult female mice 48 h after hCG injection following superovulation. P4 concentrations were measured using a commercial ELISA kit.

For serum analysis, blood samples were collected in 1.5 mL centrifuge tubes either from the ophthalmic venous plexus or via cardiac puncture under anesthesia. After standing at room temperature for 15 min, the samples were then centrifuged at 3000 rpm for 20 min to separate the serum. The supernatant serum was carefully transferred to fresh centrifuge tubes and stored at −80 °C for subsequent hormone analysis.

For ovaries hormone measurements, ovaries collected during the diestrus stage were weighed, and homogenized in an appropriate volume of PBS. Hormone concentrations were normalized to ovarian weight.

Levels of FSH, LH, E2 and P4 were measured using commercial ELISA kits (Mlbio, ml001910 for FSH, ml063366 for LH, ml063198 for E2 and ml058395 for P4), following the manufacturer’s instructions.

### 2.6 RNA isolation and quantitative reverse transcription-PCR (qRT-PCR) analysis

Total RNA was extracted from whole ovaries using RNAiso PLUS (TaKaRa 9109) following the manufacturer's instructions. Reverse transcription was performed using the 5 × All-In-One RT MasterMix (ABM G490) to synthesize complementary DNA (cDNA). qRT-PCR was conducted on a QuantStudio 5 Real-Time PCR System (Applied Biosystems A28575) using TB Green Premix Ex Taq II (Tli RNase H Plus) (TaKaRa RR820A) as previously described [17].

Primer sequences used for amplification are provided in Table S1. Gene expression levels were normalized to internal controls (*18S* rRNA or *Rpl19*) and calculated using the 2−ΔΔCT method. Data are presented as the average fold change ± standard error of the mean (SEM) from at least three biological replicates.

### 2.7 RNA-Sequencing (RNA-seq) and analysis

MII oocytes were harvested from the oviducts following superovulation. Cumulus cells were removed via hyaluronidase treatment, and total RNA was extracted using the Arcturus PicoPure RNA isolation kit (Applied Biosystems KIT0204). RNA-seq libraries were constructed using the KAPA Stranded RNA-Seq Kit for Illumina (Roche KK8440), and 150-bp paired-end sequencing was performed on an Illumina HiSeq 2500 platform at Berry Genomics Corporation.

Raw sequencing data were subjected to quality control using FastQC (v0.11.5). Adapter sequences and low-quality reads were removed using BBDuk (v38.34). Clean reads were aligned to the mouse genome (mm9) using HISAT2 (v2.1.0), and alignment results were processed into BAM format with Samtools. Gene expression was quantified as fragments per kilobase of transcript per million mapped reads (FPKM) using StringTie (v2.0.4). The resulting “.bam” and “.bai” files were visualized in the integrative genomics viewer (IGV) to assess genome-wide expression patterns and confirm KO genotypes.

For downstream transcriptomic analysis, the expression matrix was imported into R software. Principal component analysis (PCA) was conducted using the prcomp function to evaluate clustering of biological replicates. Visualization of PCA results was performed using the ggplot2 package.

Differential gene expression (DGE) analysis was conducted using the limma package. Differentially expressed genes (DEGs) were identified based on the following criteria: FPKM > 1, |fold change (FC)| > 1.5 and *p*-value < 0.05. Due to the limited number of significant DEGs detected using false discovery rate (FDR)-adjusted *p*-value (adj.P-value), raw *p*-values were used as the selection threshold. Identified DEGs were further categorized into upregulated, downregulated, or unchanged groups.

To visualize DGE profiles, volcano plots were generated using ggplot, and heatmaps were constructed with the heatmap.2 function. Functional enrichment analysis, including Gene Ontology (GO) and pathway enrichment, was performed using the Database for Annotation, Visualization and Integrated Discovery (DAVID). Additional gene function annotation was performed through the Metascape platform to identify key biological processes associated with DEGs.

### 2.8 Statistical analysis

All data are presented as mean ± SEM. Statistical significance was determined using appropriate tests, and *p*-values less than 0.05 were considered statistically significant.

## 3 Results

### 3.1 Loss of Obox1 leads to subfertility in female mice

**Fig. 1:**
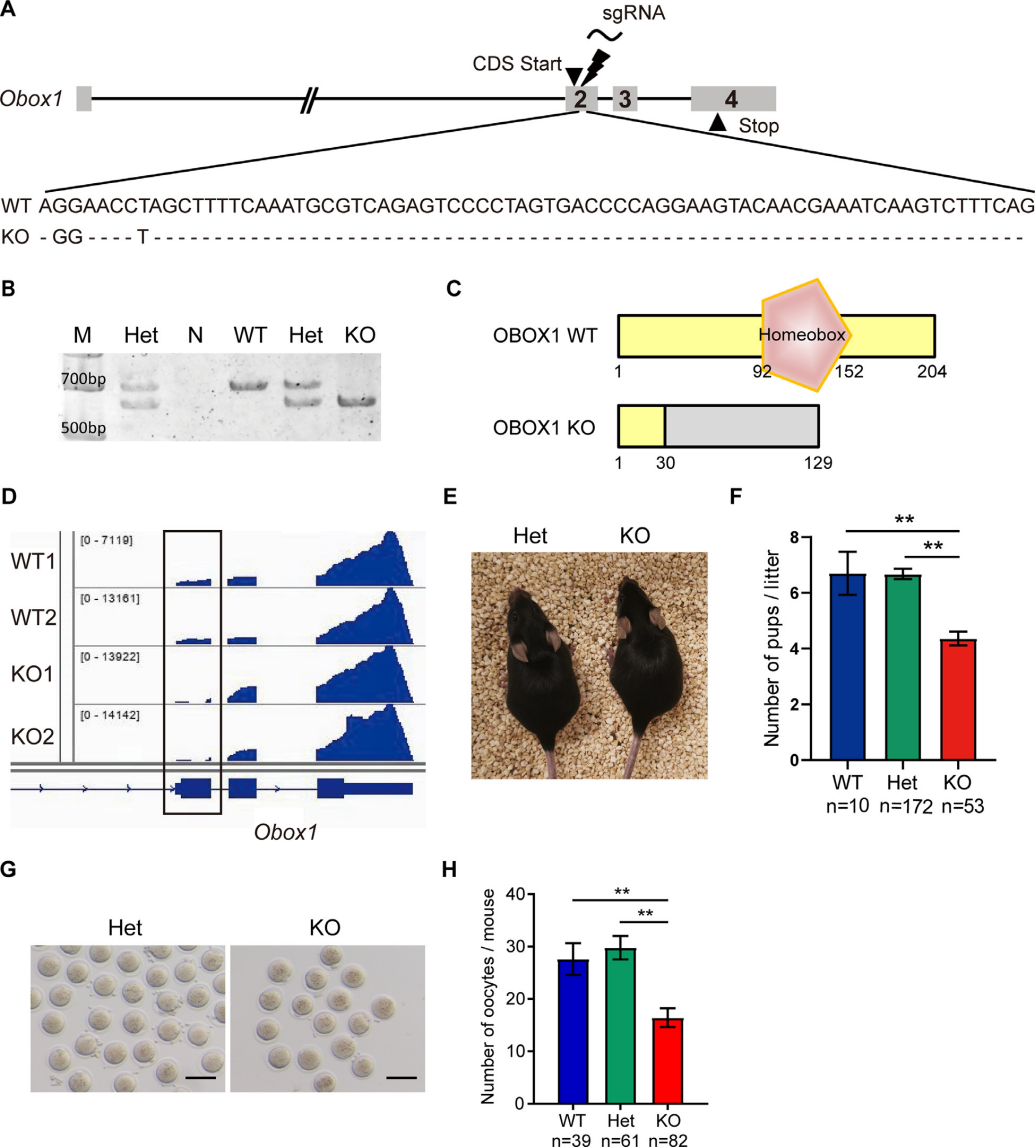
**Deletion of *Obox1* Leads to Subfertility in Female Mice.** See also Fig. S1 and S2. A. CRISPR-Cas9 targeting strategy used to generate *Obox1* knockout mice. A 73-bp deletion combined with a 3-bp insertion in exon 2 results in a net 70-bp indel, disrupting the coding sequence. B. Genotyping results showing wild-type (WT), heterozygous (Het) and homozygous knockout (KO) alleles. M, DNA marker; N, negative control. C. Schematic comparison of OBOX1 protein sequences between WT and KO mice. The homeobox domain is indicated in brown; shared sequences are highlighted in yellow. D. IGV browser view of *Obox1* RNA-seq reads in MII oocytes. In WT oocytes continuous read coverage is observed across exon 2. In KO oocytes, reads mapping to the targeted region within exon 2 are absent, confirming successful transcript disruption. E. Representative images of adult female *Obox1* Het and *Obox1* KO mice. F. Litter sizes produced by WT, *Obox1* Het, and *Obox1* KO females. Data are presented as means ± SEM. ^⁎⁎^*p* < 0.01 by Student’s *t*-test. G. Morphology of MII oocytes collected from adult *Obox1* Het and KO mice after superovulation. Scale bars, 100 µm. H. Quantification of MII oocytes collected from adult WT, *Obox1* Het and KO females after superovulation. Data are presented as means ± SEM. ^⁎⁎^*p* < 0.01 by Student’s *t*-test.

To investigate the role of *Obox1* in oogenesis and folliculogenesis, we employed CRISPR-Cas9 technology to introduce indels around exon 2 of the *Obox1* gene, which contains the start of the coding sequence (CDS). This genome editing introduced a compound mutation consisting of a 73-base pair (bp) deletion and a 3-bp insertion in exon 2, resulting in a net loss of 70 bp (Fig. 1A, 1B). The indel caused a frame-shift mutation that disrupted the open reading frame from the 31st amino acid onward, abolishing the conserved homeodomain region (Figs. 1C and S1). Due to the high sequence similarity among OBOX family proteins, the available antibodies could not distinguish between OBOX1 and OBOX2. However, transcriptome analysis of MII oocytes confirmed the deletion of exon 2 in *Obox1* KO mice (Fig. 1D).

Loss of *Obox1* did not affect overall mouse viability or survival (Fig. 1E). However, female KO mice exhibited impaired fertility, as evidenced by a nearly 50% reduction in average litter sizes compared to age-matched WT or Het females, regardless of the males’ genotype (Fig. 1F).

To further assess the effect of *Obox1* deficiency on oocyte yield, we quantified MII oocyte following superovulation in *Obox1* KO mice at different ages. In adult females, the number of oocytes retrieved from KO mice was reduced to less than 60% of that in WT or Het mice (Fig. 1G, H). This reduction was even more pronounced in pubertal *Obox1* KO mice (Fig. S2A, S2B). Despite the decrease in quantity, KO oocytes displayed normal morphology, comparable to WT or Het mice (Figs. 1G and S2A).

Together, these findings demonstrate that *Obox1* is dispensable for mouse viability, but essential for optimal female fertility, as its deficiency results in reduced oocyte yield and subfertility.

### 3.2 Obox1 deficiency does not affect early embryonic development

**Fig. 2:**
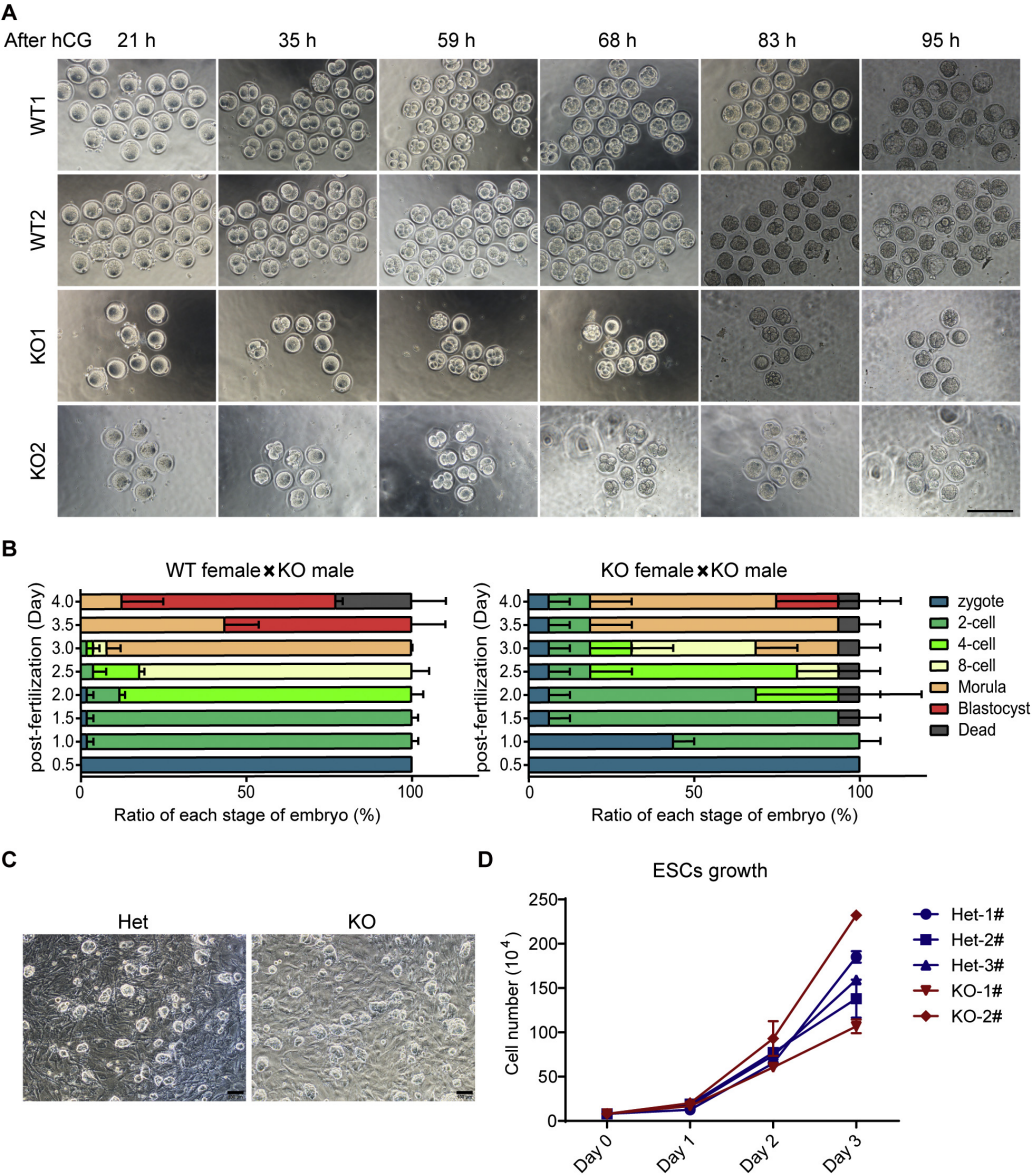
***Obox1* Deficiency Does Not Affect Early Embryonic Development.** See also Fig. S3. A. Representative images showing embryonic development after ICSI using MII oocytes from WT or *Obox1* KO mice and sperm from *Obox1* KO mice. Embryos were monitored at various stages. Scale bars, 200 µm. B. Quantification of embryos at different developmental stages after ICSI using MII oocytes from WT or *Obox1* KO mice and sperm from *Obox1* KO mice. Data are presented as percentages of total embryos observed at each stage. C. Morphology of embryonic stem cells (ESCs) derived from *Obox1* Het and KO blastocysts. ESC colonies were cultured on mitomycin C-treated mouse embryonic fibroblasts and observed under phase-contrast microscopy. Scale bars, 100 µm. D. Growth curve of ESCs derived from *Obox1* Het and *Obox1* KO blastocysts. Cell proliferation was assessed by manual cell counting over a defined culture period. Data are presented as mean ± SEM.

To evaluate the developmental potential of *Obox1* deficient oocytes, we performed *in vitro* fertilization (IVF) or intracytoplasmic sperm injection (ICSI) using MII oocytes collected form WT and *Obox1* KO females, and sperm derived from *Obox1* KO males. The resulting embryos, either *Obox1* Het or homozygous KO, were cultured *in vitro* for subsequent developmental assessment. Compared to their Het counterparts, *Obox1* KO embryos exhibited a slight delay in cleavage-stage progression (Figs. 2A, B and S3A, S3B). While the majority of Het embryos reached the 4-cell stage, a large proportion of KO embryos remained at the 2-cell stage. Similarly, when Het embryos developed to the 8-cell stage, KO embryos were predominantly at the 4-cell stage.

Despite this developmental lag, *Obox1* KO embryos were able to reach the blastocyst stage and successfully undergo hatching (Fig. S3A, S3B). To further evaluate the developmental competence and quality of the blastocysts, we derived ESCs and assessed their morphology and proliferative capacity. ESCs derived from *Obox1* KO blastocysts exhibited comparable colony morphology and growth kinetics to those derived from Het or WT embryos (Fig. 2C, D).

These results suggest that although *Obox1* deficiency may transiently delay early embryonic cleavage events, it does not impair the ability of embryos to reach the blastocyst stage or to generate ESCs with normal growth potential.

### 3.3 Obox1 KO mice exhibit reduced hormone levels at diestrus

**Fig. 3:**
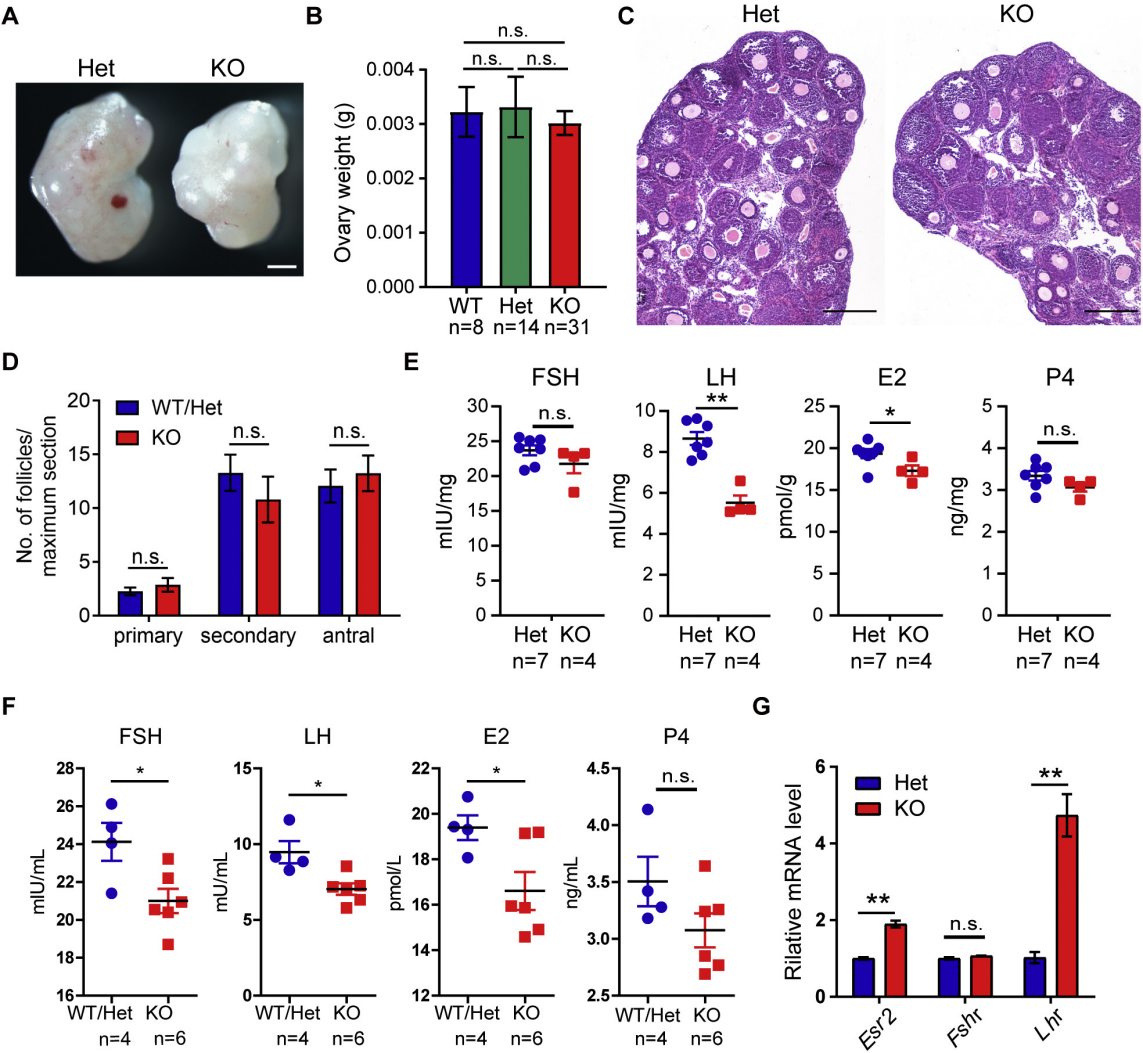
***Obox1* KO Mice Display Abnormal Hormone Levels at Diestrus.** A. Representative photographs of ovaries from WT and *Obox1* KO females. Scale bars, 500 µm. B. Ovarian weight of adult WT (*n* = 8), *Obox1* Het (*n* = 14), and *Obox1* KO (*n* = 31) females. Data are presented as mean ± SEM. n.s., not significant (*p* > 0.05) by Student’s *t*-test. C. Hematoxylin and eosin (H&E) staining of ovaries from 3-week old WT/Het and *Obox1* KO females at diestrus. Scale bar, 200 μm. D. Quantification of follicles at various developmental stage based on H&E staining of ovarian sections from adult WT/Het and *Obox1* KO females. Statistical analysis was performed on the number of follicles per maximum section (WT/Het: *n* = 9; KO: *n* = 8). Data are presented as the mean ± SEM. n.s., not significant (*p* > 0.05) by Student’s *t*-test. E. Enzyme-linked immunosorbent assay (ELISA) measurements of ovarian follicle-stimulating hormone (FSH), luteinizing hormone (LH), estrogen (E2) and progesterone (P4) in WT, Het, and KO females at diestrus. Data are presented as the mean ± SEM. n.s., not significant (*p* > 0.05), *p < 0.05, **p < 0.01 by Student’s *t*-test. F. ELISAs showing the serum levels of FSH, LH, E2 and P4 in WT, Het, and KO females at diestrus. Data are presented as the mean ± SEM. n.s., not significant (p > 0.05), *p < 0.05 by Student’s *t*-test. G. Quantitative reverse transcription PCR (qRT-PCR) analysis of mRNA expression levels of estrogen receptor beta (*Esr2*), FSH receptor (*Fshr*) and LH receptor (*Lhr*) in ovaries from *Obox1* Het and KO females. Data has normalized to *18S* rRNA and are presented as the means ± SEM. n.s., not significant (*p* > 0.05), ^⁎⁎^*p* < 0.01 by Student’s *t*-test.

Given the reduced ovulation efficiency observed in *Obox1* KO females, we further examined ovarian morphology and hormone levels. Although some *Obox1* KO ovaries appeared slightly smaller than those from WT or Het littermates (Fig. 3A), there was no statistically significant difference in ovarian weight among the groups (Fig. 3B). Histological analysis of ovaries collected at diestrus stage revealed no significant difference in follicle numbers compared to controls (Fig. 3C, D).

Despite the relatively normal ovarian histology, hormone assays revealed a marked endocrine deficiency in *Obox1* KO mice. ELISA analysis showed that LH levels within ovarian tissue reduced by nearly 65% in *Obox1* KO ovaries (Fig. 3E). Serum levels of FSH, LH and E2 were significantly reduced in *Obox1* KO females compared to controls (Fig. 3F).

Interestingly, this hormonal insufficiency was accompanied by a compensatory upregulation of hormone receptor expression. Specifically, mRNA levels of LH receptor (*Lhr*) and estrogen receptor beta (*Esr2*) were significantly elevated in *Obox1* KO ovaries (Fig. 3G), suggesting potential feedback regulation in response to reduced systemic hormone levels.

In summary, *Obox1* deficiency leads to significantly lower circulating and intra-ovarian levels of gonadotropins (FSH and LH) and steroid hormone E2 during diestrus, indicating a critical role for *Obox1* in maintaining endocrine homeostasis.

### 3.4 Abnormal ovulation and luteinization in Obox1 KO mice

**Fig. 4:**
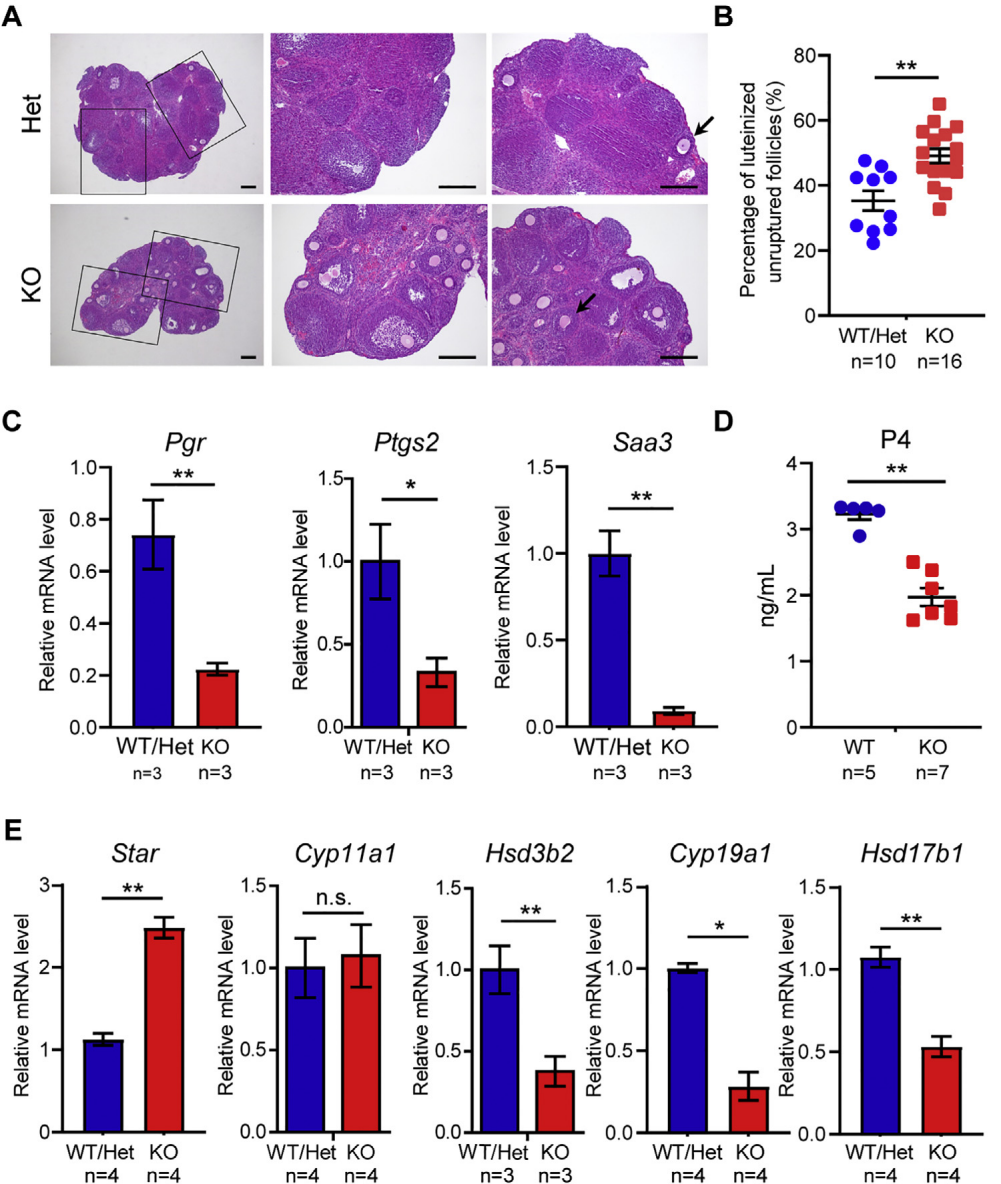
**Defective Ovulation and Steroidogenesis in *Obox1* KO Ovaries after Superovulation.** See also Fig. S4. A. Representative H&E-stained ovaries from 3- to 4-week-old *Obox1* WT/Het and KO mice at 14-16 h post-hCG injection following superovulation. Arrows indicate unruptured follicles. Scale bar, 200 μm. B. Quantification of the percentage of the unruptured follicles after hCG treatment in *Obox1* Het and KO female mice. Data are presented as mean ± SEM. ^⁎⁎^*p* < 0.05 by Student’s *t*-test. C. qRT-PCR analysis of ovulation-induced genes expression, including *Pgr, Ptgs2*, and luteal-related gene *Saa3*, in *Obox1* WT/Het and KO ovaries at 48 h after hCG treatment. Gene expression was normalized to *Rpl19*. Data are presented as the means ± SEM. *p < 0.05, ^⁎⁎^*p* < 0.01 by Student’s *t*-test. D. ELISA showing serum P4 level in *Obox1* WT and KO females 48 h after hCG treatment. Data are shown as the means ± SEM. ^⁎⁎^*p* < 0.01 by Student’s *t*-test. E. qRT-PCR analysis of steroidogenic gene expression in *Obox1* WT/Het and KO ovaries 48 hours after hCG treatment. Expression was normalized to *Rpl19*. Data are presented as means ± SEM. n.s., not significant (*p* > 0.05), *p < 0.05, **p < 0.01 by Student's *t*-test.

Since the significant reduction in LH levels observed in *Obox1* KO ovaries, we hypothesized that subfertility in these mice may be attribute to ovulation dysfunction. To test this, we performed superovulation by administering hCG to mimic the physiological surge of LH, allowing us to assess the capacity of *Obox1* KO females to respond to LH-induced ovulation. Both *Obox1* Het and KO mice developed large antral follicles 46–48 h after PMSG injection, indicating normal follicular development prior to ovulation (Fig. S4A). However, 16 h post-hCG administration, while most follicles in WT or Het mice had ovulated, a significant proportion of follicles in *Obox1* KO ovaries remained unruptured (Fig. 4A, B). Despite exogenous hCG supplementation, the number of ovulated oocytes in *Obox1* KO mice remained significantly lower than in WT/Het controls, suggesting that mimicking LH function alone is insufficient to fully restore ovulation in *Obox1*-deficient females.

To assess corpus luteum formation, we examined the ovaries of 5-week-old mice. In *Obox1* KO mice, the number of corpus lutea was significantly reduced compared to controls at this age (Fig. S4B). This result implies that, in addition to reduced LH levels, intrinsic ovarian or follicular defects might also contribute to the subfertility phenotype observed in *Obox1* KO mice.

LH surge-induced ovulation is mediated by the transcriptional activation of key genes in granulosa cells, including progesterone receptor (*Pgr*) and prostaglandin-endoperoxide synthase 2 (*Ptgs2*). PGR, transducing P4 signaling, mediates ovulation transcription through RUNX transcription factor interactions and chromatin remodeling [23,24], while *Ptgs2*, also known as cyclooxygenase-2 (COX-2), catalyzes the conversion of arachidonic acid to prostaglandins that are essential for follicle rupture, corpus luteum formation, ovarian blood flow, and hormonal regulation [25]. Another gene, serum amyloid A3 (*Saa3*), known to be associated with high-density lipoprotein (HDL) that transports cholesterol to gonads, was localized exclusively to newly formed corpora lutea and drastically reduced in granulosa cells isolated from ovaries that fail to ovulation [26]. To further investigate the impact of *Obox1* KO on the transcription of these ovulation-related genes, we measured their expression in ovarian post-superovulation. The expression levels of these genes were markedly downregulated in *Obox1* KO ovaries (Fig. 4C), suggesting defective transcriptional activation during ovulation and luteinization.

The primary physiological function of the corpus luteum is the secretion of progesterone [27]. To further evaluate the effects of *Obox1* deficiency on corpus luteum function, we examined serum P4 levels after superovulation. The serum P4 level in *Obox1* KO mice was significantly lower than those in WT controls (Fig. 4D).

Steroid hormones production within ovarian follicles is critical for successful ovulation and subsequent luteal function. In granulosa cells, LH signaling through luteinizing hormone/choriogonadotropin receptors (LHCGRs) promotes the expression of key steroidogenesis enzymes, such as steroidogenic acute regulatory protein (StAR), cholesterol side-chain cleavage enzyme (CYP11A1) and 3β-hydroxysteroid dehydrogenase/delta5 delta4-isomerase (HSD3B1 or HSD3B2), which mediate the initial steps of steroid hormone synthesis [28,29]. After ovulation, the granulosa-lutein cells exhibit suppressed expression of cytochrome P450 family 17 subfamily a member 1 (CYP17A1), which converts P4 into 17α-hydroxyprogesterone and subsequently into androstenedione (a precursor of estrogens), thus limiting this conversion and maintaining high P4 levels.

To further elucidate the role of *Obox1* deficiency in luteal steroidogenesis, we examined the expression of key P4 biosynthetic enzymes in ovaries 48 h post-hCG treatment. During early luteal formation, *Star* is dramatically induced in the theca cells and mediates cholesterol delivery to the inner mitochondrial membrane for de novo steroidogenesis. Within mitochondria, CYP11A1 converts cholesterol to the first steroid hormone pregnenolone, which is then converted to P4 by HSD3B. In *Obox1* KO ovarian, the expression level of *Star* was significantly increased, *Cyp11a1* remained unchanged, while *Hsd3b2* were markedly decreased after superovulation (Fig. 4E). The dysregulation in the expression of key P4 synthesis enzymes likely underlies the diminished P4 production observed after ovulation in *Obox1* KO mice.

In addition, we examined the expression of enzymes involved in cholesterol and estrogen synthesis. 17β-hydroxysteroid dehydrogenase type 7 (HSD17B7), which catalyzes the final step of converting 7-dehydrocholesterol into cholesterol, a precursor for steroid hormone, was upregulated in *Obox1* KO ovary (Fig. S4C), suggesting that reduced steroid hormones levels may not be due to substate deficiency. In contrast, enzymes involved in E2 biosynthesis, including cytochrome P450 family 19 subfamily a polypeptide 1 (CYP19A1), which converts androgens into estrogens, and 17β-hydroxysteroid dehydrogenase type 1 (HSD17B1), which converts estrone (E1) to estradiol (E2), were downregulated (Fig. 4E). This expression pattern provides a molecular explanation for the reduced E2 levels observed in *Obox1* KO mice and further underscores the essential role of OBOX1 in regulation of steroid hormone biosynthesis, particularly E2 production.

### 3.5 Transcriptomic dysregulation of Obox1 KO MII oocytes

The ovulation defects and dysregulation of steroidogenic enzymes observed in *Obox1* KO mice all require the involvement of ovarian somatic cells or occur within them. Given that OBOX1, functions as an oocyte-specific transcription factor, we sought to investigate how it might regulate the function of ovarian somatic cells. To address this question, we performed transcriptome sequencing of MII oocytes isolated for superovulated, age-matched adult WT and KO females. Total RNA from pooled oocytes was extracted and used for RNA-seq library construction.

**Fig. 5:**
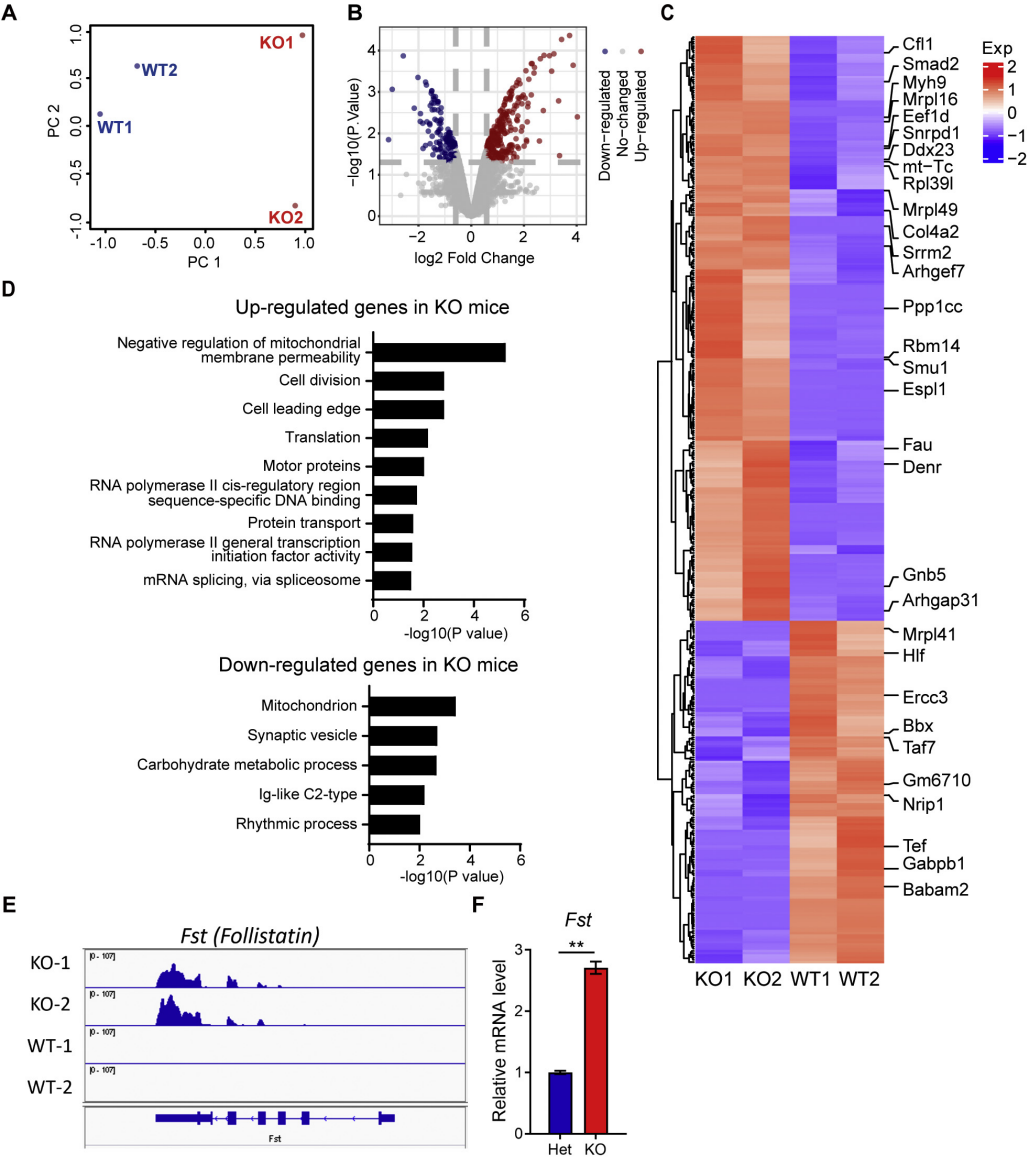
**Transcriptomic Abnormalities in MII oocytes from *Obox1* KO Mice.** See also Fig. S5 and Table S2. A. Principal component analysis (PCA) of RNA-seq data showing the global transcriptomic differences between WT and *Obox1* KO MII oocytes. B. Volcanic plot displaying differentially expressed genes (DEGs) in MII oocytes from *Obox1* KO mice compared with WT controls. C. Heatmap showing expression profiles of DEGs in MII oocytes (FPKM > 1, fold change > 1.5, *p* value < 0.05). D. Gene Ontology (GO) enrichment analysis of genes dysregulated in *Obox1* KO MII oocytes. E. IGV browser view of RNA-seq reads showing expression of *Fst* in MII oocytes from WT and *Obox1* KO mice. F. qRT-PCR analysis of *Fst* mRNA levels in ovaries from *Obox1* Het and KO females (*n* = 3). Data are normalized to *18S* rRNA and presented as the means ± SEM. ^⁎⁎^*p* < 0.01 by Student’s *t*-test.

Bioinformatics analysis revealed the transcriptomic features of *Obox1* KO MII oocytes. Principal component analysis (PCA) showed distinct clustering between KO and WT groups (Fig. 5A). We used FPKM > 1, fold change > 1.5, and *p* value < 0.05 as the criteria for differential expression gene (DEG) analysis. The volcano plot indicated a higher number of upregulated genes than downregulated genes in KO oocytes (Fig. 5B). Compared to WT, *Obox1* KO MII oocytes exhibited 280 upregulated and 128 downregulated genes (Fig. 5C; Table S2), with approximately one-third of these DEGs identified as non-coding RNAs. GO enrichment analysis revealed that the upregulated genes were enriched in pathways related to cytoplasmic dynein motor proteins (*Dynlt1f, Dynlt1b, Dynlt1c, Dynlt1a*), cell division (*Mis18a, Men1, Kifc1*), cell leading edge (*Pxn, Cfl1, Dpp9*), endocytic recycling (*Snx31, Gga3, Ehd1*), transcription (*Taf6l, Tfap4, Ep400, Tasp1*), mRNA splicing (*Srrm2, Smu1, Rbm14, Snrpd1*) and translation (*Fau, Mrpl49, Mrpl16, Rpl27, Rpl39l*). In contrast, downregulated genes were enriched in mitochondrion function (*Fads6, Eci2, mt-Tm, mt-Tq, Coq6, Gabpb1*), vesicle and cell projection (*Rab3d, Slc6a17, Arcn1, Slc22a17, Arcn1*), and DNA repair and cell cycle (*Babam2, Nme5, Ercc3, Anapc4, Fzr1, Tex50*), signaling transduction (*Lrrc24, Ptprm, Mrgprb3, Vmn1r129, Grm3, Armc2, Mpp6*), RNA processing and transcription regulation (*U2af1l4, Rbm5, Tef*) (Fig. 5D; Table S2).

The downregulation of genes related to energy metabolism, nucleotide metabolism involved in DNA replication, and cell cycle could explain the slight delay in early embryonic development observed in *Obox1* KO embryos. At the same time, the upregulation of genes involved in material transport, cell migration, transcription, and translation suggests enhanced molecular exchange and signaling activity between KO oocytes and their surrounding somatic cells.

Previous studies reported high *Obox1* expression in oocytes of *Gdf9*-deficient mice [16]. We therefore investigated the expression of key paracrine and regulatory factors in *Obox1* KO ovaries by qRT-PCR. Compared to WT/Het controls, KO ovaries showed upregulation of *Gdf9* and *Bmp15*, both encode oocyte-secreted TGF-β superfamily ligands essential for folliculogenesis and granulosa cell function [30] (Fig. S5A). Interestingly, the transcription factor germ cell nuclear factor (GCNF/NR6A1), which directly represses *Gdf9* and *Bmp15* expression, was also upregulated in KO ovaries (Fig. S5A). This is consistent with phenotypes observed in *Gcnf*-conditional KO mice (*Gcnf* ^fl/fl^; *Zp3*-Cre), which exhibit altered steroidogenesis and subfertility [31].

RNA level of FOXL2, a key transcription factor for granulosa cell proliferation and differentiation, was also elevated in KO ovaries (Fig. S5A) [32]. MOV10, a dosage-sensitive RNA helicase known to repress LINE1 retrotransposition in oocytes, was downregulated in *Obox1* KO oocytes but upregulated in the whole ovary (Table S2; Fig. S5A), suggesting a possible compensatory mechanism in somatic cells[3].

Notably, we also detected upregulation of *Notch2* and *Jag1* in KO ovaries, indicating enhanced NOTCH signaling between oocytes and surrounding somatic cells (Fig. S5A). This reciprocal signaling is known to be crucial for oocyte-somatic cell communication and follicular development [4]. In contrast, RNA level of TAF4B, a gonadal-enriched TFIID subunit essential for meiosis and oocyte competency, was downregulated in KO ovaries [33].

Collectively, these findings suggest that *Obox1* regulates the oocyte transcriptome in a manner that affects paracrine signaling to somatic cells. In particular, its loss may enhance NOTCH pathway activation and alter GDF9 and BMP15-mediated follicular regulation.

To further explore the influence of *Obox1* on the HPO axis, we examined transcriptomic changes in follistatin (FST), a known modulator of gonadotropin signaling. IGV visualization of RNA-seq data revealed upregulation of *Fst* in *Obox1* KO oocytes (Fig. 5E), which was further validated by qRT-PCR in whole ovaries (Fig. 5F). Follistatin acts by binding and antagonizing activins and BMPs, thereby modulating follicular development, oocyte maturation, and luteinization, and the feedback regulation of FSH. Its upregulation in *Obox1* deficient ovaries provides a plausible mechanism by which oocyte-derived signals alter gonadotropin responsiveness and ovulatory outcome.

## 4 Discussion

The oocyte-specific expression pattern of *Obox1* suggests its involvement in female reproduction [16]. In this study, we generated *Obox1* KO mice and observed reduced fertility. At the diestrus stage, *Obox1* KO mice exhibited significantly decreased levels of both circulating and intra-ovarian steroid and gonadotropic hormones, with the most pronounced reduction observed in LH. Administration of exogenous hCG during superovulation, intended to mimic LH activity, failed to rescue the reduced ovulation phenotype in OBOX1 KO mice. Even after superovulation, the number of ovulated oocytes in adult *Obox1* KO females was approximately half that observed in WT or Het controls. Although the developmental potential of these oocytes was largely unaffected, corpus luteum formation and progesterone secretion following ovulation were significantly compromised. These findings suggest that the *Obox1* deficiency may result in ovulatory dysfunction.

Ovulatory events are initiated by a midcycle surge of LH. hCG, an functional LH analog, binds to the LH/hCG receptor with a higher affinity than LH and is commonly used to trigger ovulation in both animal models and clinical fertility treatments [34]. In *Obox1* KO mice, we observed significantly reduced LH levels and administered exogenous hCG to simulate LH function. However, this intervention did not rescue the ovulation defect, suggesting that the ovulation insufficiency in *Obox1* KO mice may not be solely attributable to hormonal dysregulation.

Transcriptome analysis revealed upregulation of the *Fst* in KO oocytes, which may interfere with BMP and other signaling pathways essential for follicular development. At the ovarian level, we also detected elevated expression of *Fst* and genes involved in the NOTCH signaling pathway. These alterations may contribute to disrupted gonadotropin-steroid hormone feedback and impaired follicle maturation.

Ovarian follicular development and ovulation are intricately regulated by the HPO axis [35]. In *Obox1* KO MII oocytes, we observed a significant upregulation of *Fst*, a key regulator of TGFβ signaling. Follistatin exerts its function by selectively binding to TGFβ family ligands, particularly activin, and inhibiting downstream signaling [36]. Through this mechanism, follistatin antagonizes the FSH-promoting effects of activin and acts as a negative regulator of FSH secretion, contributing to the maintenance of hormonal homeostasis within the HPO axis [37].

This balance between activin and follistatin is crucial for normal follicular development: while activin promotes follicle progression, follistatin prevents overstimulation. Follistatin is typically expressed at low levels in pre-antral follicles, where it can inhibit aromatase and inhibin secretion, while promoting P4 synthesis, there supporting luteinization [38]. As the follicles mature and a dominant follicle emerges, activin production declines while follistatin and inhibin levels increases [39].

Beyond its endocrine functions, follistatin also modulates ovarian stromal cell activity, corpus luteum function, and oocyte quality by influencing TGF-β signaling, apoptosis, and mitochondrial dynamics. Dysregulated follistatin expression has been implicated in reproductive disorders such as polycystic ovary syndrome (PCOS) and premature ovarian insufficiency [40].

In addition to postnatal roles, follistatin also contributes to primordial germ cell formation and differentiation during embryogenesis, and facilitates embryo-maternal communication during implantation.

Taken together, these findings suggest that OBOX1 may regulate FSH biosynthesis and secretion in the pituitary via the follistatin-mediated modulation of TGFβ family signaling. This mechanism provides a potential explanation for the subfertility phenotype observed in *Obox1* KO mice.

## 5 Conclusion

In summary, our study demonstrates that loss of *Obox1* results in reduced litter size and impaired ovulation. As an oocyte-specific transcription factor, OBOX1 plays a pivotal role in regulating ovarian somatic cell function by modulating the paracrine secretion of key oocyte-derived factors such as follistatin, GDF9, and BMP15. These factors collectively influence steroidogenesis and female fertility. However, whether OBOX1 directly binds to the promoters of these target genes remain to be elucidated in future studies.

## Data availability

The RNA sequencing data and associated analyses generated in this study have been deposited in the NCBI’s Gene Expression Omnibus (GEO) under accession number GSE207448.

## Declaration of competing interest

The authors declare that they have no conflicts of interest in this work.

## Appendix Supplementary materials

Image, application 1

Image, application 2
