# Supplementary material for: Obox1 deficiency impairs fertility in female mice
Source: Fundam Res. 2025 Apr 19;5(4):1570–80. doi: 10.1016/j.fmre.2025.04.008 (PMC12327860; doi:10.1016/j.fmre.2025.04.008)
Supplement: Supplementary file 1 [file mmc1.docx]

**
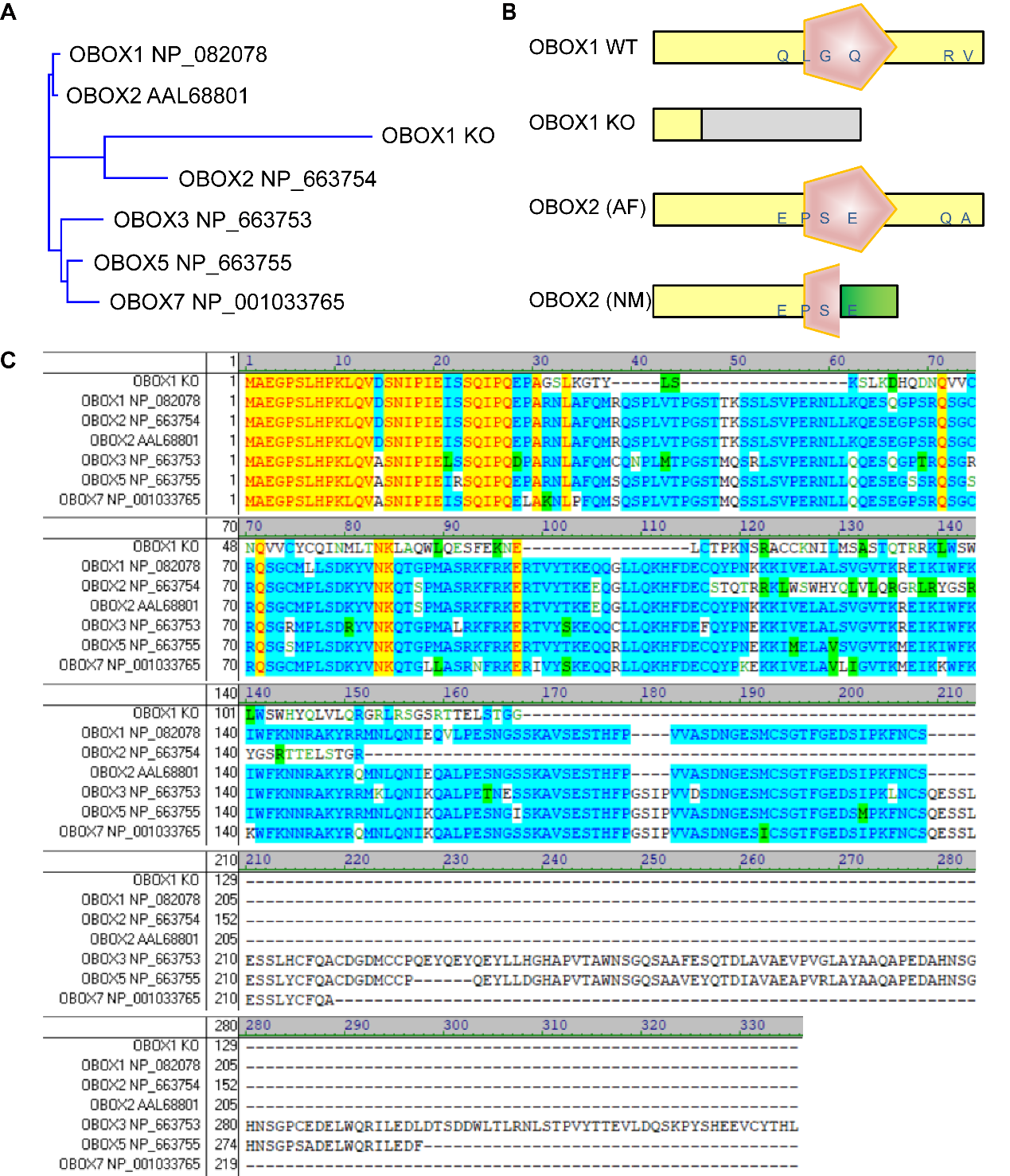
Figure S1. Alignment of *Obox1* KO and *Obox* Family Members in Protein Sequences. Related to Figure 1.**

1. Phylogenetic tree of OBOX family proteins constructed based on amino acid sequences using alignment software. The accession numbers of the protein sequences used are: OBOX1 WT (NP_082078), OBOX1 KO (in this study), OBOX2 (NP_663754 and AAL68801), OBOX3 (NP_663753), OBOX5 (NP_663755) and OBOX7 (NP_001033765).
2. Schematic representation of OBOX1 and OBOX2 protein structures. Protein sequences used include: OBOX1 WT (NP_082078), OBOX1 KO (in this study), and OBOX2 isoforms, AF (AF461107) (AAL68801) and NM (NM_145708) (NP_663754). OBOX1 and OBOX2 (AF) share 97% identity, differing at 6 amino acid residues. OBOX2 (NM) harbors a frameshift mutation that results in a truncated homeobox domain. In *Obox1* KO, a frameshift mutation disrupts the coding region starting from the 31st amino acid. Schematic symbols: brown pentagon, homeobox domain; yellow rectangle, identical sequence; green rectangle, frameshift sequence in OBOX2 (NM); grey rectangle, frameshift in OBOX1 KO.
3. Protein sequence alignment of OBOX family members. In *Obox1* KO, an indel mutation introduced a frameshift that disrupts the coding sequence staring from the 31^st^ amino acid. Sequences conservation among OBOX family members is illustrated using color coding: identical residues are shown as red text on a yellow background, highly conserved residues are highlighted with a blue background, and similar amino acid resides are indicated by green text.


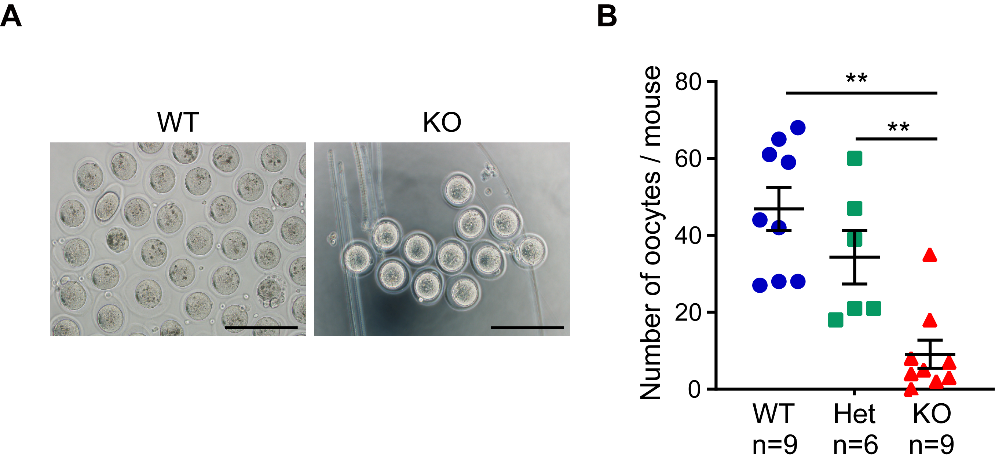


**Figure S2. Reduced Oocyte Ovulation in pubertal *Obox1* KO mice. Related to Figure 1.**

1. Morphology of the MII oocytes collected from 3-week-old WT, *Obox1* Het and *Obox1* KO mice after superovulation. Scale bars, 200 µm.
2. Quantification of MII oocytes collected from 3-week-old WT, *Obox1* Het and *Obox1* KO mice after superovulation. Data are presented as means ± SEM. ***p* < 0.01 by Student’s *t* test.


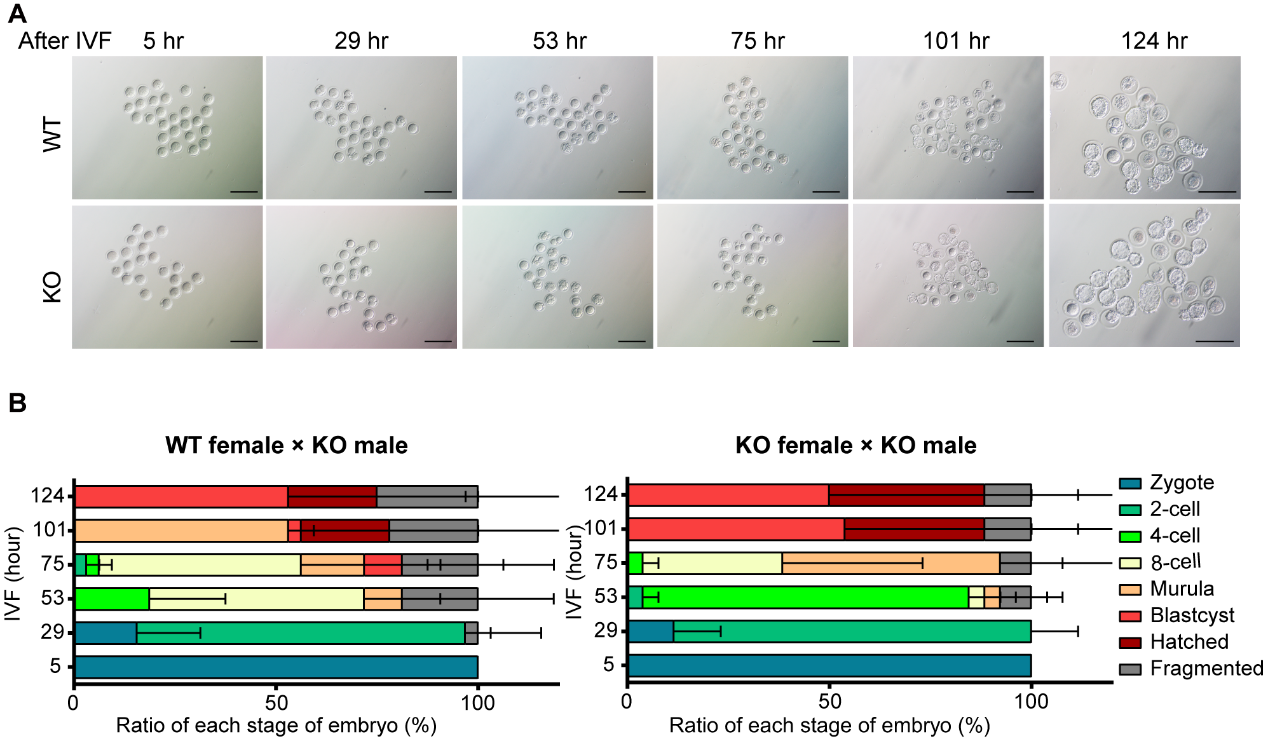


**Figure S3. *Obox1* Deficiency Does Not Affect Early Embryonic Development. Related to Figure 2.**

1. Representative images showing embryonic development following in vitro fertilization (IVF) using MII oocytes from WT or *Obox1* KO adult female mice and s perm from adult male *Obox1* KO mice. Embryos were monitored at various stages post-IVF. Scale bars, 200 µm.

# Quantification of embryos at different developmental stages after IVF using MII oocytes from WT or *Obox1* KO mice and sperm form *Obox1* KO mice. Data are presented as percentages of total embryos observed at each stage.

**
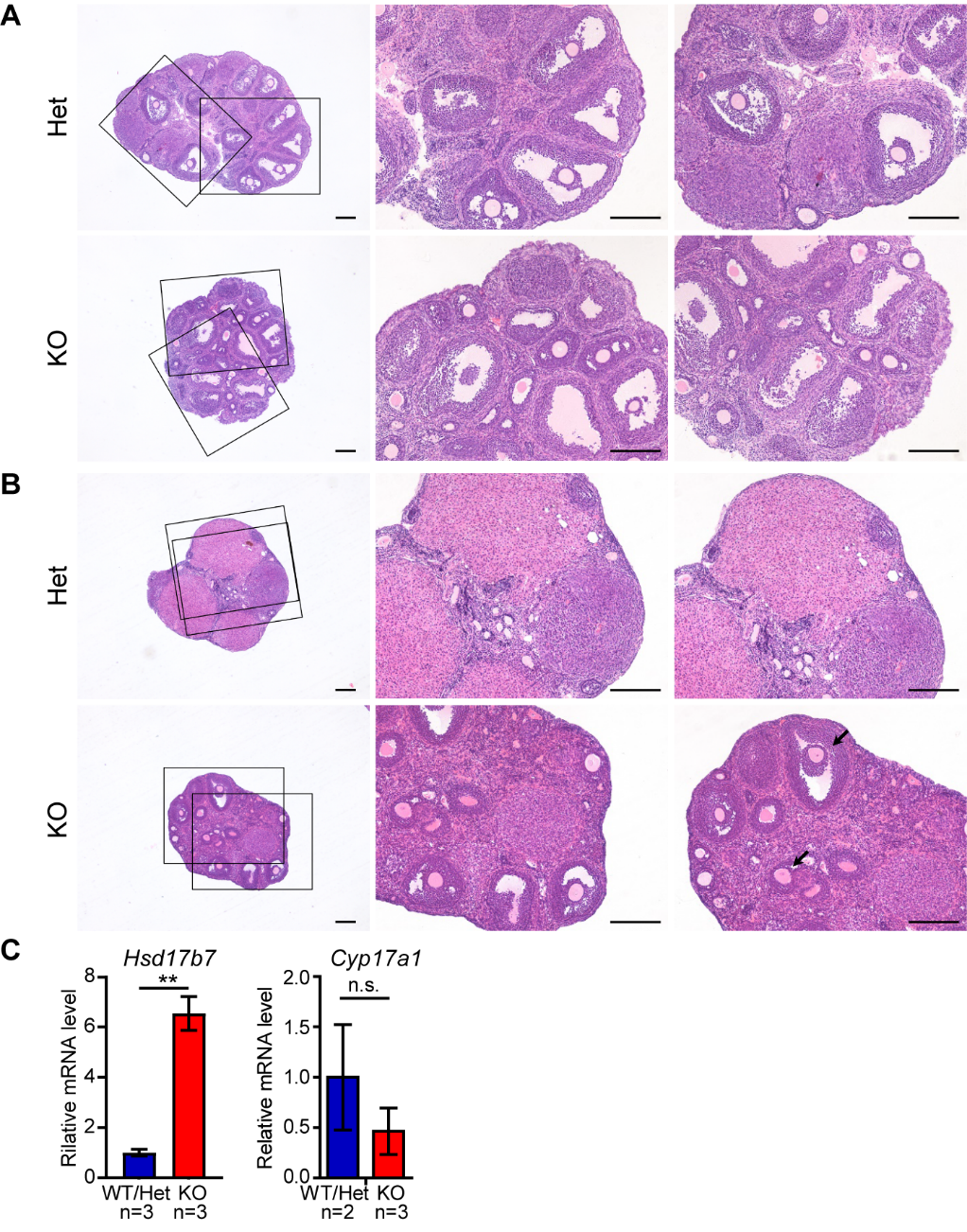
**

**Figure S4. Defective Ovulation and Steroidogenesis in *Obox1* KO Ovaries after Superovulation. Related to Figure 4.**

1. Representative H&E-stained ovaries sections from 3- to 4-week-old *Obox1* Het and KO female mice collected 46 hours after PMSG injection. Scale bar, 200 μm.
2. Representative H&E-stained images of ovaries from 5-week-old *Obox1* Het and KO females. Arrows indicate unruptured follicles. Scale bar, 200 μm.
3. qRT-PCR analysis of steroidogenic gene expression in *Obox1* WT/Het and KO ovaries 48 hours after hCG injection. Gene expression was normalized to *Rpl19*. Data are presented as means ± SEM. n.s., not significant (*p* > 0.05), ***p* < 0.01 by Student’s *t* test.

**
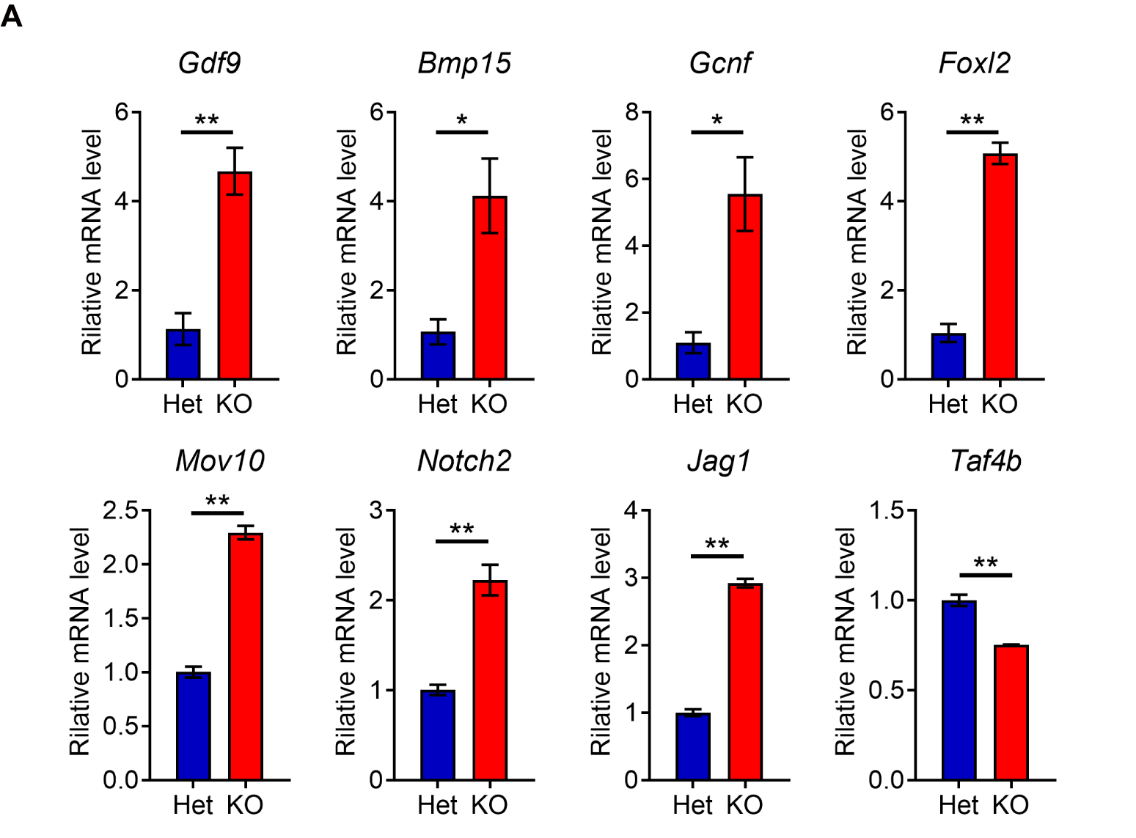
**

**Figure S5. Abnormal Expression of Folliculogenesis and Oogenesis Genes in *Obox1*-null Ovaries. Related to Figure 5.**

1. qRT-PCR analysis of folliculogenesis-related genes, including growth differentiation factor 9 (*Gdf9*), bone morphogenetic protein 15 (*Bmp15*), germ cell nuclear factor (*Gcnf*), forkhead box L2 (*Foxl2*), DEAD-box RNA helicase molt-related virus 10 (*Mov10*), Notch receptor 2 (*Notch2*), Jagged canonical Natch ligand 1 (*Jag1*) and TATA-box binding protein associated factor 4B (*Taf4b*) in *Obox1* Het and *Obox1* KO ovaries. Gene expression was normalized to *18S* rRNA. Data are presented as means ± SEM. **p* < 0.05, ***p* < 0.01 by Student’s *t* test. n=3.

**Table S1. Primer Sequences Used in this Paper. Related to Figure 1/3/4/5/S4/S5.**

| **[1]Table S1. Primer Sequences Used in this Paper. Related to Figure 1/3/4/5/S4/S5.** | | |
| --- | --- | --- |
| **Primer Name** | **Primer sequence** | **Reference** |
| Pair-KO-sgRNA 1-1 | TGAAAAGCTAGGTTCCTTGC |  |
| Pair-KO-sgRNA 1-2 | CAGAGTCCCCTAGTGACCCC |  |
| Pair-KO-sgRNA 2-1 | TGATTTCGTTGTACTTCCTG |  |
| Pair-KO-sgRNA 2-2 | CTACTTAAGCAAGAGTCTCA |  |
| *Obox1* genotyping F | GCAGAATTTGCCTGCCTGAAAGAC |  |
| *Obox1* genotyping R | CGTATTTATTGACTTGGATGCCCTGAGAG |  |
| *Esr2* rt F | AAACAGAGAGACCCTGAAGAGAA | [2] |
| *Esr2* rt R | CCTCTTGGCGCTTGGACTAG |  |
| *Fshr* rt F | CCAACCTACCCAACTTGCATG | [3] |
| *Fshr* rt R | CAGATATCGGAGACTGGGAAGATT |  |
| *Lhr* rt F | CGCTTTCCAAGGGATGAATA | [1] |
| *Lhr* rt R | CTGGAGGGCAGAGTTTTCAG |  |
| *18S* rt F | CCGCGGTTCTATTTTGTTGG | [4] |
| *18S* rt R | GGCCGTCCCTCTTAATCATG |  |
| *Pgr* rt F | CATGGTCCTTGGAGGTCGTAA | origene |
| *Pgr* rt R | CTCTCGTTAGGAAGGCCCAC |  |
| *Ptgs2* rt F | CACCTCTGCGATGCTCTTCC | [5] |
| *Ptgs2* rt R | CTGGTCAAATCCTGTGCTCATAC |  |
| *Saa3* rt F | GTTGACAGCCAAAGATGGGT | [5] |
| *Saa3* rt R | GATGACTTTAGCAGCCCAGG |  |
| *Star* rt F | GTGCTTCATCCACTGGCTGGAA | origene |
| *Star* rt R | GTCTGCGATAGGACCTGGTTGA |  |
| *Hsd3b2* rt F | ATCAGGGTCCTGGACAAGGTCT | origene |
| *Hsd3b2* rt R | TGGCAAGCTCTCCTCAGGTACT |  |
| *Cyp19a1* rt F | CGAAGCAGCAATCCTGAAGGAG | origene |
| *Cyp19a1* rt R | CCAAGTCCACAACAGGCTGGTA |  |
| *Hsd17b1* rt F | AGGTGACGGAGCTCTTCTTG | origene |
| *Hsd17b1* rt R | CGACATAGCTGCTGCCACT |  |
| *Hsd17b7* rt F | GGGCCAAAAGATGGACATAG | origene |
| *Hsd17b7* rt R | AGGAACGCCTACATCAGCTC |  |
| *Cyp11a1* rt F | TGCTCAACCTGCCTCCAGACTT | origene |
| *Cyp11a1* rt R | ACTGGCTGAAGTCTCGCTTCTG |  |
| *Cyp17a1* rt F | TTTATGCCTGAGCGCTTCTT | origene |
| *Cyp17a1* rt R | GCAGCAAGGCCATGAAGATA |  |
| *Rpl19* rt F | TCATCCGCAAGCCTGTGACTGT | origene |
| *Rpl19* rt R | ACCTTCTCAGGCATCCGAGCAT |  |
| *Gdf9* rt F | GATGGGACTGACAGGTCTGG | origene |
| *Gdf9* rt R | CAGCGGTCCTGTCACCTG |  |
| *Bmp15* rt F | AAGGGAGAACCGCACGATTG | origene |
| *Bmp15* rt R | TGCTTGGTCCGGCATTTAGG |  |
| *Gcnf* rt F | TCATCAAGCGGAGTTTACCC | origene |
| *Gcnf* rt R | CCCCACAGATGAGACAGGTT |  |
| *Foxl2* rt F | ACAACACCGGAGAAACCAGAC | origene |
| *Foxl2* rt R | CGTAGAACGGGAACTTGGCTA |  |
| *Mov10* rt F | CCCGGTGAAAGCTATGAACT | origene |
| *Mov10* rt R | GTTTCTGCTCCTTCTGCTCC |  |
| *Notch2* rt F | GCTGTCAATAATGTGGAGGCG | [6] |
| *Notch2* rt R | TTGGCCGCTTCATAACTTCC |  |
| *Jag1* rt F | TGGATTCAAGTGTGTGTGCC | [7] |
| *Jag1* rt R | GGAAGGCAATCACAGTAGTAGC |  |
| *Taf4b* rt F | GATGTTACTAAAGGCAGCCAAGAGT | [3] |
| *Taf4b* rt R | CTGCTCTGGATCTTCTTTATTGGAG |  |
| *Fst* rt F | TACTGTGTGACCTGTAATCGGA | [3] |
| *Fst* rt R | TGATACACTTTCCCTCATAGGCT |  |

1. K. Wilson, J. Park, T. E. Curry, Jr. *et al*, Core Binding Factor-β Knockdown Alters Ovarian Gene Expression and Function in the Mouse. Mol Endocrinol. 30 (2016) 733-747. <http://doi.org/10.1210/me.2015-1312>

2. J. R. Wardell, K. M. Hodgkinson, A. K. Binder *et al*, Estrogen responsiveness of the TFIID subunit TAF4B in the normal mouse ovary and in ovarian tumors. Biol Reprod. 89 (2013) 116. <http://doi.org/10.1095/biolreprod.113.111336>

3. E. Voronina, L. A. Lovasco, A. Gyuris *et al*, Ovarian granulosa cell survival and proliferation requires the gonad-selective TFIID subunit TAF4b. Dev Biol. 303 (2007) 715-726. <http://doi.org/10.1016/j.ydbio.2006.12.011>

4. P. P. Law, L. A. Mikheeva, F. Rodriguez-Algarra *et al*, Ribosomal DNA copy number is associated with body mass in humans and other mammals. Nat Commun. 15 (2024) 5006. <http://doi.org/10.1038/s41467-024-49397-5>

5. S. A. Robertson, I. Christiaens, C. L. Dorian *et al*, Interleukin-6 is an essential determinant of on-time parturition in the mouse. Endocrinology. 151 (2010) 3996-4006. <http://doi.org/10.1210/en.2010-0063>

6. A. Turbic, S. Y. Leong, A. M. Turnley, Chemokines and inflammatory mediators interact to regulate adult murine neural precursor cell proliferation, survival and differentiation. PLoS One. 6 (2011) e25406. <http://doi.org/10.1371/journal.pone.0025406>

7. M. Prat, K. Coulson, C. Blot *et al*, PPARγ activation modulates the balance of peritoneal macrophage populations to suppress ovarian tumor growth and tumor-induced immunosuppression. J Immunother Cancer. 11 (2023) <http://doi.org/10.1136/jitc-2023-007031>
